# Supplementary material for: Are carbon emissions trading and green financial instruments synergistic? -Comprehensive quantitative research based on content analysis
Source: PLoS One. 2024 Mar 7;19(3):e0298601. doi: 10.1371/journal.pone.0298601 (PMC10919601; doi:10.1371/journal.pone.0298601)
Supplement: S1 Data — (ZIP) [file pone.0298601.s001.zip › Data/地级市Z_F工作报告.zip!/╜¡╬≈/╔╧╚─╩╨/└┤╫╘╣1⁄2╓┌║┼í╛┬φ┐╦╩2╛▌═°í┐_2006.docx]

各位代表：

现在，我代表市人民政府向大会作工作报告，并就《上饶市国民经济和社会发展第十一个五年规划纲要(草案)》作出说明，请予审议，并请各位市政协委员和列席会议的同志提出意见。

一、“十五”时期工作回顾

过去五年，全市人民在中共上饶市委的领导下，坚持以邓小平理论和“三个代表”重要思想为指导，全面贯彻和落实科学发展观，紧紧围绕“建设四省交界区域中心城市和快速发展地区”的战略目标，开拓创新，团结拼搏，改革开放和现代化建设取得显著成就，“十五”计划全面超额完成。

(一)综合经济实力显著增强。全市生产总值由2000年的173.4亿元增加到2005年的388.1亿元，年均增长13.6%，比“九五”平均增幅高6.4个百分点；人均GDP由2000年的343美元增加到2005年的739美元，GDP总量和人均GDP实现五年翻一番。财政总收入由2000年的15.7亿元增加到2005年的33.6亿元，年均增长16.4%，比“九五”平均增幅高9.5个百分点，五年也实现翻一番。全社会固定资产投资由2000年的32.8亿元增加到2005年的227亿元，年均增长47.2%，比“九五”平均增幅高40.8个百分点，五年累计完成622.6亿元，是“九五”时期的2.9倍。社会消费品零售总额由2000年的81.3亿元增加到135.8亿元，年均增长10.8%，比“九五”平均增幅高1.9个百分点。金融机构存款余额380.7亿元，贷款余额296.5亿元，分别比"九五"期末增长121.6%和86.6%，贷款余额和贷款增量均居全省第2位。

(二)经济结构调整迈出大步。三次产业结构由2000年的32.7：29.2:38.1调整到2005年的21.8:42.5:35.7，五年内二产上升13.3个百分点。工业主导型经济增长格局基本形成，工业增加值占GDP比重30.9%，五年提高6.1个百分点；财政总收入中有55.2%来自工业，五年提高6个百分点；工业经济效益综合指数达到122.4%。工业园区已成为吸纳就业的主渠道、招商引资的新平台、带动经济快速发展的增长极，园区面积36.1平方公里，入园企业总数1169个，安置就业突破9万人，年销售收入达160亿元，全省10个销售收入过30亿元园区上饶占有2个；机械、建材、食品、有色金属、纺织服装和医药化工等六大支柱产业销售收入占全市规模以上工业的72.7%；规模以上工业企业达到389家，其中年销售收入过亿元的企业33家，比2000年增加25家；凤凰光学仪器有限公司的“凤凰”、月兔集团的“月兔”商标荣获中国驰名商标，凤凰光学仪器有限公司的单镜头反光相机、透视取景机荣获“中国名牌产品”称号，20个产品获“江西名牌产品”称号。农业的基础地位更加巩固，农业总产值135亿元，年均增长6.5%，高于“九五”时期4.1个百分点。粮食总产52.4亿斤，创历史最高水平；经济作物产值占农业总产值比重达56.9%，比2000年提高11.2个百分点；拥有绿色（有机）食品212个，总数列全省第一位；农业企业由2000年的900家发展到2005年的2216家，其中规模以上农业企业由2000年的66家发展到2005年的254家，国家、省、市三级龙头企业达99家；万年贡米、广丰白耳黄鸡、横峰葛获地理标志产品保护，建有国家级农业标准化示范区7个、省级农业标准化示范区8个。以旅游业为主导的第三产业快速发展，拥有国家重点风景名胜区2个、国家4A级景区4个、国家森林公园5个，婺源县被列为全国生态文化旅游示范县，2005年全市旅游总人数超过570万人次，旅游总收入达32.5亿元；商贸物流、房地产、金融保险、邮电通讯等服务业加快发展，第三产业增加值138.7亿元，年均增长14.1%，高于"九五"平均增幅2.9个百分点。

(三)基础设施建设提速推进。高速公路建设从无到有，建成高速公路174.8公里，在建高速公路206.2公里，高速公路通车里程占全省的11%；建成快速通道1135公里，比“九五”时期增加492公里；硬化和改造农村公路12200公里，县到乡实现油(水泥)路化、乡到村油(水泥)路率达50%。大型电力项目建设从无到有，有史以来投资最大的黄金埠电厂开工建设，50万伏输变电工程已经启动,新建22万伏变电站4座、11万伏输变电站10座，农村电网改造顺利完成，城网改造正在抓紧实施，实现了城乡居民生活用电同网同价。大型商品粮基地建设从无到有，投资7500万元的国家商品粮基地项目落户上饶，实施了移民建镇、退田还湖、平垸行洪、鄱阳湖二期综合治理、城市防洪以及农业综合开发、土地整理、以工代赈和老区扶贫工程，完成了463座病险水库除险加固，更新改造了滨湖地区的机电排灌设施，改造中低产田81万亩。建设了政务信息网络平台,实现了省、市、县纵向联通。开通了市政府门户网站"中国上饶"和市长热线电话，完成了农村电话村村通工程，固定电话用户达100.3万户、移动电话用户达107.1万户、宽带互联网用户达19.7万户。

(四)城乡区域发展更加协调。抓住撤地设市契机，加速推进中心城市建设，累计完成城建投资175亿元，编制各项规划51项，实施了45项城建重点工程，完成了324条里弄小巷改造，市区建成区面积由撤地设市前的17平方公里拓展到35.2平方公里，市区人口由撤地设市前的17万人增加到35万人，人均绿化面积由撤地设市前的4.52平方米扩大到7.1平方米。坚持分类指导原则，出台了《加快滨湖地区发展的决定》和《加快城镇化进程的决定》，有力推动了县域经济发展，上饶、广丰、玉山、信州、弋阳、横峰、铅山等七县（区）"半小时经济圈"初步形成，鄱阳、余干、万年三县经济总量占全市份额加大，婺源、德兴两县（市）经济特色更加鲜明，12个县(市、区)财政收入均过亿元，其中过7亿元1个、过4亿元1个、过2亿元6个，广丰县成为全省经济发展综合先进县，余干、上饶两县分别进入全省农业、工业发展先进县行列。进一步实施了"大乡大镇"战略，有31个乡镇财政收入过千万元，德兴市泗洲镇成为上饶市第1个财政收入过亿元乡镇；有26个乡镇挤进全省百强乡镇，总数列全省第2位；加大了撤乡并村和村镇规划力度，乡镇总数已由撤地设市前的287个减少到188个，村委会总数由原来的2938个减少为2085个，所有的乡镇都完成了总体规划编制，848个行政村完成村庄规划编制。全市城市化水平由2000年的18.6%上升到2005年的27%，年均提高1.9个百分点。

(五) 体制机制创新取得突破。五年来，我们按照完善社会主义市场经济体制的要求，深入推进了各项改革。先后实施了农村税费改革、农村义务教育体制改革、林业产权制度改革、水利工程产权制度改革和粮食流通体制改革，取消了“三提五统”、农村教育集资和除烟叶外的农业特产税，免征了农业税，实现了农民合同内"零"负担。初步建立了国有资产管理、监督、营运体系，组建了国有资产管理委员会和工业国有资产经营公司，完成了市本级企业国有资产的清产核资。出台了鼓励全民创业的“三十条”措施，民营经济快速发展，城镇以上固定资产投资中来自非国有单位投资占57.9%，五年提高30.1个百分点；民营经济占GDP比重33.9%，五年提高15.8个百分点，多种经济成分共同发展的格局基本形成。政府机构改革和行政审批制度改革稳步展开，为加强行政服务和环境建设，组建了市经济发展服务中心；为加强宏观经济调控，将市物价局并入了市发改委，市中小企业局并入了市经贸委，市招商局、市外贸局、四省九市联络处合并组建了市外经贸委；为加强城市规划管理，组建了市城市规划局和市城市管理局；为加强旅游资源的规划、开发和建设，组建了市旅游局和上饶集中营名胜区、方志敏革命根据地以及铜钹山、葛仙山、鹅湖山风景名胜区管委会；为加强电子政务，组建了市信息化工作办公室。初步建立了多元化的投融资机制，组建了市城建投资公司和交通投资集团，鑫新股份成功上市。进行了国库集中支付、部门综合预算、“乡财县管”等各项财政管理体制改革，协调推进了金融、流通、社会保障、科技教育、文化卫生等领域的改革。

(六)大开放主战略深入实施。提出了“掉头向东、通江达海、对接长珠闽”的开放取向，加快了与长珠闽地区对接，加速融入全球化进程，积极参与武汉经济协作区、福州经济协作区、赣浙闽皖四省九市经济协作区及“泛珠三角”区域经济合作，加大招商引资力度，不断拓展对外交流与合作的空间。五年累计直接利用外资4.3亿美元、实际引进市外资金586亿元，分别是“九五”时期的13.8倍和20.7倍；2005年引进合同资金亿元以上项目50个,总投资73.9亿元；工业项目506个，总投资140.3亿元。外贸出口总额由2000年的0.3亿美元增加到2005年的1.3亿美元，年均增长30.8%，五年翻了两番多。

(七)社会各项事业和谐发展。五年来，我们始终把发展社会事业放在更加重要的位置，促进经济建设与社会事业协调发展。科教兴市战略深入实施，2005年专利申请获准量115件；取得科技成果32项，其中省级8项，有2项获省科学技术三等奖；新增民营科技企业31家、省级以上高新技术企业5家，有3项高新技术项目获国家科技创新基金支持。义务教育得到提升，优质高中建设力度加大，高中等教育资源进一步整合，民办和职业教育有较快增长，上饶师专升格为上饶师院，并在此基础上着手组建上饶学院，组建了上饶职业技术学院。疾病预防控制和突发公共卫生事件医疗救助体系得到健全，新型农村合作医疗试点工作顺利推进，乡镇和社区卫生服务网络初步建立。深入开展了文化“四进社区”、“三下乡”等活动，审定公布了首批95处市级文物保护单位，赣剧《詹天佑》、歌曲《清贫颂》、舞蹈剧《铁窗英魂》、儿童剧《我们的网络妈妈》、原生态民歌《呼牛调》等一批文艺精品获得省以上奖励。广播电视事业荣获全省八连冠，完成了422个村的广播电视“村村通”工程，组建了上饶人民广播电台和广播艺术歌舞团，有线电视用户达到52.4万户。成功举办首届市运会和10多次全国性体育赛事，余干籍运动员朱智福在韩国釜山亚运会上夺得金牌，于莎莎在第十届全运会上荣获冠军，为江西和上饶争得了荣誉。人口低生育水平保持稳定，生育质量和人口素质有新的提高。“生态上饶”建设取得实效，森林覆盖率达到57.7%，主要河流断面水质优于Ⅲ类比例达96.3%，其中信江达100%，城市环境空气质量达到国家二级标准，优良率为100%。社会治安综合治理力度加大，“四五”普法取得成效，建立了困难群众的生活保障机制、社会矛盾的排查调处机制、突发性公共事件的应急处理机制和社会治安防控体系。认真贯彻新信访条例，妥善处理人民群众来信来访，突出解决了农村征地、城镇房屋拆迁、企业重组改制、农民工工资拖欠等涉及群众切身利益的问题。生产安全、交通安全、防火安全和食品安全进一步加强，安全生产形势总体平稳。群众性精神文明创建活动扎实开展，城镇“五城”同创和农村“五个一”创建活动卓有成效，荣获全国民族团结进步模范集体和全省社会治安综合治理先进设区市，市区创建省级文明、卫生、园林城市目标已经实现，全社会文明程度提高，城乡居民安全感增加。新闻出版、地方志、档案、保密、外事侨务、涉台事务、气象服务、防震减灾、民兵预备役和"双拥"工作、国防教育、人民防空、社会福利、妇女儿童、老龄、残疾人等事业健康发展。

(八) 城乡居民生活明显改善。城镇居民人均可支配收入由2000年的4891元增加到2005年的8629元，首次超过全省平均水平，年均增长12%；农民人均纯收入由2000年的2038元增加到2005年的3226元，年均增长9.6%，与全省平均水平的差距由2000年的97元缩小到2005年的37元。城乡居民人均住房面积分别达到21平方米和35平方米，五年分别增加7.5平方米和6.2平方米。全面实施积极的就业政策，社会保障体系进一步完善，五年累计新增城镇就业18.6万人，解决21.3万人饮水困难，2005年有23.5万人参加养老保险、24.6万人参加医疗保险、27.3万人参加失业保险、12万人享受城市居民最低生活保障、17万人得到农村特困户救助、22.8万贫困学生享受"两免一补"资助、9000名库区、湖区、深山区群众实施移民扶贫搬迁。

(九)政府行政效能不断提高。认真执行市人大及其常委会的决议，坚持向市人大及其常委会报告工作并接受监督；重视和支持人民政协以及各民主党派、工商联履行政治协商、民主监督和参政议政职能；认真办理人大代表建议、议案和政协委员提案，办结率每年均达到100%。强化基层民主法制建设，全面实施《行政许可法》，落实行政执法责任制，深化行政审批制度改革，综合行政执法工作顺利开展。进一步强化了对市委决策的落实力度，对重点工程、重点项目、重点工作进行专项督查；开展了年度主要经济工作巡视督查活动，加大了经济工作考核力度。充分发挥监察、审计等部门的监督作用，落实党风廉政建设责任制，推进政务公开，开展行风评议活动，政务环境明显改善。

各位代表！“十五”发展成就来之不易。省委、省政府和中共上饶市委的坚强领导，为上饶这艘快速前进的航船把握了正确航向；全市人民贡献智慧，挥洒汗水，众人划桨，为上饶快速发展提供了强大动力。在此，我代表市人民政府，向战斗在各条战线的全市人民和驻饶人民解放军、武警官兵、公安干警，向给予政府工作大力支持的人大、政协及各界人士，向广大老干部、老同志和关心家乡发展的在外乡友，向为上饶发展作出贡献的建设者，向所有参与、支持和关心上饶发展的海内外朋友表示衷心的感谢和崇高的敬意！ 五年的发展成就令人鼓舞，五年的奋斗历程难以忘怀。在工作中，我们深切体会到，坚持以人为本、富民优先，任何时候都不能忘记；坚持加快发展、科学发展，任何时候都不能动摇；坚持开拓奋进、创新克难，任何时候都不能削弱；坚持求真务实、真抓实干，任何时候都不能懈怠。但我们必须清醒地看到，上饶仍处在“爬坡上坎、负重前进”的发展阶段，还面临许多亟待解决的矛盾和问题。主要是：自主创新能力不够强，经济增长方式比较粗放，经济发展与环境保护的矛盾日益突出；工业项目规模较小、档次较低，产业集聚效应还没有显现出来；农业产业化程度不够高，农业增长、农民增收的任务还相当艰巨；社会保障压力还比较大，一些群众的生活还比较困难；政府职能转变有待加强，依法行政水平有待进一步提高。对待这些问题，我们应有强烈的忧患意识、责任意识和竞争意识，认真加以解决。

二、“十一五”时期发展的总体思路

各位代表，经过“十五”的发展，上饶经济社会已经站在新的历史起点上。未来五年，是建设四省交界区域中心城市和快速发展地区全面推进的重要时期。从国际形势看，世界经济将继续保持恢复性增长态势，经济全球化、区域经济一体化和世界范围的产业结构调整和产业转移步伐加快，为我市进一步扩大开放提供了重要契机。从国内形势看，科学发展观全面贯彻落实，我国经济社会发展进入新阶段，社会主义市场经济体制逐步完善，加入世贸组织的效应进一步显现，扩大内需成为长期战略，为我市的发展注入新的外部动力；尤其是党中央、国务院把“中部崛起”和社会主义新农村建设作为国家发展的重大战略，更是我市加快发展、科学发展、协调发展的重要机遇。从全省发展格局看，省委、省政府已把上饶作为区域发展的重点城市，对上饶的发展寄予厚望，提出了一系列明确要求，为我市实现新一轮跨越式发展指明了方向。面临良好的发展机遇和光明的发展前景，站在新的起点上谋划今后五年的发展，我们对未来更加充满信心。根据《中共上饶市委关于制定国民经济和社会发展第十一个五年规划的建议》，市政府制定了《上饶市国民经济和社会发展第十一个五年规划纲要（草案）》，提交本次大会审议。《纲要（草案）》坚持以科学发展观为指导，着眼于建设四省交界区域中心城市和快速发展地区，提出了今后五年全市经济社会发展的总体要求、奋斗目标和主要任务，描绘了全市发展的宏伟蓝图和美好前景。

“十一五”时期经济社会发展的总体目标是：

--总量上台阶。经济发展速度每年保持在12.7%以上，到2010年，经济总量、人均生产总值、财政总收入比2005年翻一番，分别达到780亿元、1400美元、74亿元。

--质量大提升。到2010年，三次产业比例调整为14.7：48.1：37.2，工业增加值占生产总值比重达到37.8%，外贸出口突破5亿美元，单位生产总值能耗降低20%。

--后劲有增强。五年累计固定资产投资1750亿元，实际利用外资年均增长13.5%，基本形成快速便捷的立体交通网络和200千伏输变电主网架。城市化率每年提高2个百分点，到2010年达到37%。

--社会更和谐。城镇登记失业率控制在4.5%以内，人口自然增长率控制在8.6‰以内；森林覆盖率达到60％，城市生活污水集中处理率达到63％，生活垃圾无害化处理率达到75％。基本完成创建全国卫生城市、全国文明城市、国家园林城市、中国优秀旅游城市、全国双拥模范城市目标。

--收入快增长。城镇居民人均可支配收入年均增长8.6%、农民人均纯收入年均增长7.4%，到2010年分别达到1.3万元和4600元，城乡居民恩格尔系数下降到43％和53%以下。

“十一五”时期的发展，关键是坚持以科学发展观统领经济社会发展全局，牢牢把握“五个始终”：

第一，始终把实现又快又好发展作为“十一五”发展的主题。这既是扩大就业、提高人民群众生活水平的迫切需要，也是“十一五”时期经济工作的基本要求。为此，必须坚持发展是硬道理的战略思想，把发展作为解决一切问题的关键，聚精会神搞建设，一心一意谋发展，加快提升上饶经济实力；必须把快速发展建立在节约发展、清洁发展、安全发展、协调发展上，建立在优化经济结构、转变增长方式、提高经济运行质量和效益上。 　　 第二，始终把统筹城乡、区域协调发展作为“十一五”发展的重要原则。综合分析各县（市、区）的区位条件、发展基础、资源环境承载能力，加强分类指导，促进优势互补、良性互动、共同发展；加快调整城乡二元结构，优化人口空间布局，推动城乡资源要素合理流动，促进城市基础设施向农村延伸，城市公共服务向农村覆盖，城市现代文明向农村辐射，推进社会主义新农村建设，形成以城带乡、以工促农、城乡互动、协调发展的体制和机制；注重城市内涵式增长和质量提升，强化市区，发展县城，完善小城镇，构建分工有序、功能互补、结构协调的城镇体系。

第三，始终把做大总量、提升质量作为“十一五”发展的战略任务。突出抓好投资总量扩张，致力于推进一批基础设施项目、生产性项目和社会事业、生态建设项目投资建设，坚定不移地实施工业化核心战略，加速新型工业化进程，促进一二三产业迅速发展、经济总量迅速扩张，尽快达到在全省的应有份额；更加注重经济运行质量的提升，在壮大总量中调整优化结构、提升产业水平、增加财政收入，在壮大总量中提高城镇居民收入水平、实现农民收入持续增长，在壮大总量中加强统筹协调、走可持续发展路子。

第四，始终把改革创新、扩大开放作为“十一五”发展的根本动力。坚持推进体制机制创新，充分发挥市场机制的作用，着力形成集约型、集群型、开放型、内生型、都市型的经济形态，为上饶加快发展注入新的活力；进一步实施大开放主战略，在更广领域、更高层次上参与国际合作与竞争，全面提高对外开放水平和经济国际化程度，实现市场化、国际化与工业化、城市化、信息化的互动并进。

第五，始终把加快富民步伐、构建和谐社会作为“十一五”发展的出发点和落脚点。坚持以人为本，把富民作为优先发展的目标，调动一切积极因素，激发全社会创业活力，使人民群众的小康生活更加宽裕；坚持发展为了人民、发展依靠人民、发展的成果由人民共享，把改善民生放在突出的位置，更加注重社会发展，更加注重社会公平，突出解决好人民群众最关心、最直接、最现实的切身利益问题；坚持和谐发展，处理好改革发展稳定关系，推进民主法制建设，维护社会安全稳定，建设资源节约型、环境友好型的绿色生态上饶，使上饶的山更青、水更绿、天更蓝。

三、开好局，起好步，努力做好2006年工作

今年是实施“十一五”规划的开局之年。根据市委一届十五次全会精神，今年政府工作的总体要求是：全面贯彻党的十六届五中全会和省委十一届十次全会精神，以科学发展观统领经济社会发展全局，紧紧围绕建设"四省交界区域中心城市和快速发展地区"目标，着眼做大经济总量、增强综合实力，坚持开放带动、项目带动、科技带动，强力主攻工业，推进社会主义新农村建设，构建创新创业上饶、绿色生态上饶、和谐平安上饶，加速农业农村现代化、新型工业化、新型城镇化、经济国际化和市场化进程，努力在新的起点上实现经济社会更快更好发展。 经济社会发展的主要预期目标是：生产总值增长14%；财政总收入增长17%；全社会固定资产投资增长23.3%；社会消费品零售总额增长14%；实际引进市外资金239亿元，实际利用外资2亿美元，外贸出口2亿美元；城市化率提高2个百分点，城镇登记失业率控制在4.5%以内；城镇居民人均可支配收入增长9%，农民人均纯收入增长8%；人口自然增长率控制在8.11‰以内。 实现上述目标，要在统筹兼顾、全面推进的基础上，重点抓好以下九方面工作:

1、扎实推进社会主义新农村建设

按照“生产发展、生活宽裕、乡风文明、村容整洁、管理民主”的要求，稳步推进新农村建设。充分尊重农民意愿，坚持一切从实际出发，注重试点和分类指导，搞好乡（镇）村规划，重点支持农村公益事业建设。坚持“一看路、二看树、三看卫生、四看住”，综合整治农村环境，积极开展土地整治，继续完善农村电网，改造农村道路，发展农村沼气，建设"民富、村美、风气好"的社会主义新农村。 按照“希望在山、潜力在水、重点在田、后劲在畜、出路在工”和“品种特色化、基地规模化、生产标准化、经营产业化、投入科技化”的要求，大力发展现代农业。稳定发展粮食生产，继续执行粮食最低收购价政策，实施优质粮食产业工程，力争粮食总产稳定在52亿斤。大力发展优质、高产、高效、生态、安全农业，进一步做大做优"六大产业"，推进"一村一品"，促进优势农产品和特色农产品集中连片发展。加快推进农业产业化经营，培育和发展农副产品加工业，支持农业龙头企业做大做强。加快农业标准化建设，健全农技推广、植物保护、农产品检验检疫和动植物疫病防治体系，切实做好高致病性禽流感等重大动物疫病防控工作，尤其要严防向人传播，确保人民群众健康安全。搞好农业综合开发，提高农业综合生产能力。 按照“工业反哺农业、城市支持农村”的要求，建立以工促农、以城带乡的长效机制。进一步落实中央、省、市各项支农政策，坚持最严格的耕地保护制度，积极推进农村专业经济合作、村级集体经济股份合作，增强村级集体经济组织的服务功能，控制和化解村级债务，杜绝新的不良债务发生；加大扶贫开发力度，实施贫困村整村推进计划和深山区、库区、自然灾害频发区移民扶贫搬迁工程；加大投入力度，扩大公共财政覆盖农村范围，实行公共服务建设重点向农村倾斜；加快发展农村二、三产业，推进农村劳动力向非农产业和城镇有序转移。加强对农民职业技术和就业技能培训，培养有文化、懂技术、会管理的新型农民。

2、着力提升产业竞争力 加速推进新型工业化。

进一步加大主攻工业力度，着力提升工业园区的开发效益，依托园区建设一批特色产业基地，鼓励和引导关联企业、配套企业向重点园区集聚，抓好园区招商和入园企业的达产达标，提高园区投入强度和产出水平。着力提升支柱产业的发展水平，集中力量打造光学制造业基地、客车制造业基地、铜材精深加工业基地和新型建材业基地，促进机械、建材、食品、有色金属、纺织服装、医药化工等六大支柱产业快速增长。着力提升重点企业的创新能力，对50户成长型企业予以重点调度，支持拥有名牌产品、先进技术、资源优势、竞争力强的大企业和企业集团做大做强；抓紧在优势资源深度开发、加工工业和高新技术领域开工建设一批重大工业项目；引导和鼓励各类企业开展清洁生产，节能降耗，提高资源利用率，建设节约型工业；强化企业的自主创新能力，实施质量振兴战略，鼓励企业争创名牌。建立工业奖励制度，开展创评“先进工业县（市、区）”、“先进工业园区”、“工业十强企业”活动，新增规模以上企业100户、销售收入过亿元企业7户，努力实现“三增长、两提高、一降低”目标，即：规模以上工业增加值增长32.3%、销售收入增长31.9%、利税总额增长30.3%；工业增加值占GDP比重提高2个百分点，工业经济效益综合指数提高10个百分点；工业综合能耗降低5个百分点。 加快把旅游业培育成国民经济的重要产业。深入贯彻《关于进一步加快旅游产业发展的意见》，以三清山为龙头，以市区为中心，以婺源、龟峰、鄱阳湖为重点，策应周边景区开发，加快景区景点建设，构建"大旅游、大产业、大市场"格局。启动开发市区旅游资源，巩固发展老景区，开发建设新景区，健全旅游服务保障体系，建设游客集散中心、旅游购物中心、旅游信息服务中心、旅游教育培训中心和导游服务管理中心。建立旅游标准化体系，加大旅游创品牌力度，争取用1-2年的时间创建成“中国优秀旅游城市”、三清山申报“世界自然遗产”和龟峰申报“世界地质公园”成功；开展乡村旅游“农家乐”活动，争创全国乡村旅游示范区。强化旅游产品宣传促销，扩大上饶旅游知名度，拓展旅游市场，力争接待旅游总人次和旅游总收入分别增长30%以上。 充分发挥消费对经济的拉动作用。实施“大市场、大商贸、大物流、大集聚”战略，构建市区“丰”字形市场格局，重点抓好商贸特色街区建设，推进市区中心广场商贸超市、佳利商贸城、新火车站物流园、旭日商贸城等项目建设，完善江南商贸城、国际家具城、月亮湾汽车城、新华龙物流园等“市场集群”的服务功能，引进、培育大型国际商贸连锁企业，争取沃尔玛、雅高等国际知名企业落户上饶。进一步活跃城乡市场，继续实施“万村千乡”市场工程，搞好国家商务部日用消费品和农资“农家店”改造试点，对供销社基层网点和个体经营网点进行整合，构筑农村现代流通服务体系。进一步改善消费环境，实施放心菜、放心肉工程，培育新的消费热点，提升房地产、金融保险、中介、社区等服务业发展水平，扩大文化、教育、健身等服务性消费，促进消费需求。

3、进一步加快基础设施建设

着力推进公路交通建设，全面完成景婺黄(常)高速公路，加快建设景鹰高速公路，做好宁德至上饶高速公路启动准备工作；建设快速通道108公里，完成上乐公路路基改造工程和鄱余公路路面硬化，拓宽改造珠曹线，完成上饶至德兴、上饶至横峰两条战备公路建设，加快余干龙津大桥、鄱阳莲湖大桥建设进度；实施城乡公交一体化工程，实现市本级“半小时交通圈”内城乡公交一体化。积极配合国家完成浙赣铁路电气化改造，完成火车站主体站房建设。着力推进通讯设施建设，努力提高城乡通信网络覆盖率。进一步加强水利设施建设，继续抓好高店水库、伦潭水利枢纽建设和万年、德兴等县市城防工程的实施，继续实施鄱阳湖区机电泵站更新改造项目，完成200座病险水库除险加固工程。进一步加强电力建设，加快推进黄金埠电厂建设工程，启动50万伏输变电工程，全面改造县城电网，新建、改建22万伏输变电工程9座、11万伏输变电工程6座。

4、全面推动全民创业

深入落实全民创业“三十条”，营造更加浓厚的创业氛围。建立全民创业基金，开展创业培训，鼓励国有企业、科研院所、事业单位的工作人员依法创业，高校毕业生、退伍安置人员及下岗职工自主创业，外出务工人员返乡创业，农民进城从事二、三产业，企业家到省外、海外创业。加大财政、金融支持力度，健全信用担保体系，建立面向中小企业的技术创新、投资服务、产品检测、市场营销等公共服务平台，放宽市场准入，充分释放市内民众的投资潜力，推动民营经济快速发展和做大做强，力争来自民营经济税收占财政总收入比重提高5个百分点。

5、加快开放型经济发展 加强区域合作。

进一步扩大与长三角、珠三角、闽东南三角以及周边地区的横向联系，推动赣浙经济开发区实质性运作，更多地吸纳发达地区的资本和产业转移。 提高招商质量。坚持外资优先原则，扩大利用外资规模，继续开展重大项目招商年和“五条线”招商活动，创新招商方式, 强化企业的招商主体地位，加快“招商”向“选商”转变、“引资”向“选资”转变、“政策招商”向“环境招商”转变，着力引进一批大企业、大客商、大财团，以大项目带动产业链延伸；加强土地投入成本核算，优化引进科技含量高、吸纳就业多、环境污染少的项目；健全信息服务平台，强化跟踪服务，降低商务成本，办好第二届上饶国际投资贸易洽谈会，抓好重大招商项目调度，力争引进合同内资亿元以上项目 50 个，合同外资1000万美元以上项目 5 个。 促进外贸出口。实施多元化发展战略，优化出口商品、出口市场和贸易方式结构，鼓励具有自主知识产权和自主品牌产品的出口，在巩固提高纺织服装、农产品出口的同时，把机电产品、轻工产品的出口规模做大。加快海关的申请建设，优化投资营商环境。支持有条件的企业"走出去"，到境外投资，开发利用境外资源，拓展发展空间。

6、促进城乡区域协调发展 大力发展各具特色的县域经济。

进一步健全激励机制，赋予县级更大的发展权，激发各地创新发展的内在活力，推动各地各展所长、协调发展。推进以县城为重点的小城镇建设，把加速城镇化的注意力聚焦到完善城市功能、强化城市内涵上来，着力建设上饶、广丰、玉山、信州、弋阳、横峰、铅山等七县（区）小城市群，逐步把鄱阳、余干、广丰三县县城建成中等城市；以三清山为龙头，把玉山、婺源建成全国优秀旅游城市；打响德兴“中国铜都”品牌，将万年培育滨湖地区新型工业县，力争通过1-2年的努力，财政收入超10亿元县有1个，超5亿元县有2个以上。继续实施“大乡大镇”战略，重点支持财政收入过1000万元、镇区人口超10000人的重点镇建设，形成一批财政收入超5000万元乡镇，增强乡镇经济发展实力。继续加大村、镇规划力度，确保100%的乡镇、60%的行政村完成规划编制。 做大做强市区经济。壮大城市产业，提高上饶工业园的运作水平和质量效益，充分发挥城投公司、交投集团的作用，增强资本运作能力；加快发展城市金融保险、商业流通、饮食服务、文化娱乐等服务业。搞活城市经营，按照开放、让利、公平原则，抓好交通、污水处理、市政工程、文化、旅游等一批城市可经营项目市场化运作；进一步推进城市经营管理体制的创新，盘活城市资源，提高资源利用率。夯实产业平台，高起点修编《上饶市城市总体规划》、《上饶市土地利用总体规划》和编制各类专项规划，进一步强化规划的统一管理；高标准建设市中心区、新火车站片区，完成中心广场改造和凤凰大桥、三清山大桥、东环路、滨江路等四个连接点项目建设，推进“双塔”公园、森林公园、紫阳公园、中央公园建设，修复信江书院，着手建设新体育中心、会展中心（演出中心），启动生活垃圾处理厂、20万吨自来水厂建设和铁路新村改造；完善城市服务功能，搞好城市绿化、美化、亮化和净化，提升城市品位。

7、进一步创新体制机制

加快以乡镇机构、农村义务教育和县乡财政体制改革为主要内容的农村综合改革，进一步推进林业产权制度、水利管理体制和粮食购销市场化改革，深化土地征用、管理制度改革，切实保护基本农田。积极探索国有资源整合和利用的市场化方式，推进产权多元化改造，完善国有资产管理体制和监管机制，实现国有资产保值增值。加快建立多元化的投融资机制，鼓励民资和外资参与国有企业改组改造，鼓励社会力量、民营资本进入市政基础设施、文化、教育、医疗、体育等领域，积极探索行政事业单位经营性资产统一经营管理新模式。推进金融业改革和发展，加大利用资本市场力度，支持和鼓励各类企业改制上市，壮大企业发展规模，完成鑫新股份公司股权分置改革。进一步推进财税改革，完善公共财政体制，强化税收征管，加快县（市、区）部门预算和财政支出管理制度改革，建立政府投资项目和财政资金使用绩效评价制度。坚持政事分开，分类推进事业单位改革，促进公共事业体制转换。

8、促进各项社会事业全面发展

实施人才兴市战略，全面推行科技特派员制度，抓好全省制造业信息化示范市建设，加快构建科技创新平台。巩固和完善九年义务教育，实施农村寄宿制学校、农村远程教育、优质高中建设"三大工程"；启动上饶医学分院新校区建设，加强上饶师院、职院和电大建设，着手组建上饶学院；大力发展职业教育、成人教育和民办教育，培养多层次、技能型实用人才。着力改善医疗卫生条件，抓好婺源、广丰、玉山、弋阳、上饶县等五县的新型农村合作医疗试点，强化农村卫生、社区卫生服务，加强血吸虫病、结核病、艾滋病以及突发性重大传染性疫病的防治工作。加强公民思想道德建设，大力培育和谐文化和创新创业文化；深入开展精神文明创建活动，创建全国双拥模范城市和全国优秀旅游城市，继续实施小康文化工程、舞台精品工程和文化信息资源共享工程，加强文化基础设施建设，着手建设一批乡村多功能文化站室；整合文化资源，发展文化产业。抓紧广电中心建设和821台的搬迁，进一步实施广播电视村村通工程。大力开展群众性体育活动，抓紧筹建上饶奥林匹克中心，扎实备战全省第十二届运动会。加强人口与计划生育工作，切实降低出生人口性别比。加大环境综合整治力度，实施“绿色门槛”准入制度，全面推行清洁生产，推进绿色生态上饶建设。认真办理人民群众来信来访，积极开展法律援助，制定并实施"五五"普法规划。加强社会治安综合治理，依法严厉打击各种犯罪活动，维护社会稳定。高度重视安全生产，保障人民群众生命财产安全。重视加强国防教育，做好民兵预备役工作，增强全民国防意识。积极支持工会、共青团、妇联等群众团体开展工作，关心老龄和残疾人事业，继续做好民族宗教、外事侨务、对台、防震减灾、人防、气象、档案和地方志工作。

9、切实办好民生大事

各位代表，人民群众是我们的衣食父母。办好民生大事，是人民政府义不容辞的责任；提高人民生活水平，是政府工作的出发点和落脚点。我们将始终牢记为人民服务宗旨，始终牢记发展第一要务，始终牢记“两个务必”，时刻把人民群众的生活放在心上，把人民群众的需要作为第一追求，真心实意为人民群众办实事、办好事。实践证明，为民办事，必须办多数人受益的事，办群众最急需的事，办长远起作用的事，办力所能及的事。为此，我们郑重承诺，在改善群众生产、生活方面着力办好“十件实事”：

（1）建设社会主义新农村。市本级安排3500万元用于改善农村基础设施、整治农村生活环境，重点建设10个新农村建设示范镇、100个新农村建设示范村。

（2）创造更多的就业机会。实施农村劳动力转移培训"阳光工程"和农民科技培训，培训农村劳动力22.3万人；落实就业和再就业优惠政策，对国有下岗职工再就业扶持政策再延长三年，并扩大到城镇集体下岗职工、失地农民和退役军人，免费开展职业介绍7.4万人、再就业培训3.7万人，为困难群体（4050人员）购买政府公益性岗位1500个，确保新增城镇就业人员4.4万人，下岗失业人员再就业1.7万人，向省外输出劳务93.5万人。

（3）保障困难群众生活。市本级安排5000万元用于社会保障支出，其中180万元用于农村特困群众救助、350万元用于城市居民最低生活保障、300万元用于社会保障补助、130万元用于失业保险补助，确保离退休人员养老金按时足额发放，确保养老金社会化发放率达100%、企业退休人员社会化管理服务率达95%以上；着手建设上饶市社会救助管理站，解决流浪乞讨人员救助无场所问题。

（4）搞好扶贫开发。市本级安排455万元用于扶贫搬迁工程配套，从深山区、库区、自然灾害频发区搬迁移民8000人；解决5万农民饮水困难和饮水安全问题。

（5）建立城乡特困户医疗救助制度。市本级安排105万元用于农村大病医疗救助配套，安排420万元用于实施新型农村合作医疗试点；建立市区平价医院，确保社区卫生服务覆盖率达到80%以上。

（6）改善市区居民生活居住环境。新增城市公共绿地31万平方米，改造市区80条里弄小巷，新建10个垃圾中转站；解决困难群体的居住条件，建设经济适用房5.6万平方米。

（7）抓好农村公路建设。市本级安排800万元，配套用于农村公路建设，完成农村公路硬化1500公里，实现所有的乡镇和一半以上的行政村通油路、水泥路。

（8）支持农村教育。安排300万元用于农村学校危房改造配套；安排295万元用于农村教育"一费制"配套；安排131万元用于困难家庭学生"两免一补"配套；安排100万元用于上饶师院、上饶医专、上饶职业技术学院贫困大学生助学贷款贴息。

（9）建设食品药品放心工程。推进无公害农产品基地建设，完善食品药品检验体系，确保基地农产品抽检合格率达90%以上；县城以上推行生猪机械化屠宰，确保县城以上定点屠宰率达90%以上；对流通领域的大中型食品超市、商场和较大食品批发企业全面实行食品市场准入制度，索证索票率达95%以上；学校食堂、大中型餐饮业全面实行食品卫生监督量化分级管理；完善农村药品配送网络，确保农村药品配送率达100%。

（10）建立社会化灾害救助机制。建立和完善主要自然灾害以及重大事故的监测、预报预警系统和灾害处理应急指挥系统；推进经常性社会捐助工作网络建设和制度建设，储备足够的救援物资设备，形成一支快速反应的救灾力量，提高紧急救援能力，减少灾害造成的人员伤亡和经济损失。

四、加强政府自身建设，提高行政执行力

坚持开创性、坚韧性和操作性的统一，以更加务实、更加敬业、更加规范、更加高效的新形象、新风貌，履行职责，不辱使命。

--进一步加强法制建设。自觉接受人大及其常委会的依法监督和人民政协的民主监督，及时办理人大代表议案、建议和政协委员提案，认真听取民主党派、工商联、无党派人士和各人民团体的意见；进一步扩大公民、社会和新闻舆论对政府的监督，支持监察、审计部门依法加强政府系统内部监督。深入贯彻《行政许可法》，深化行政审批制度改革，简化行政许可程序，规范行政执法行为；全面推行行政执法责任制，严格追究执法过错责任；进一步推行政务公开，增强政府工作透明度。

--进一步创新管理机制。继续推进政府职能转变，更加注重履行社会管理和公共服务职能。改进政府管理方式，充分发挥行政规划、行政指导、行政合同的作用，进一步规范政府投资行为；完善科学民主决策机制，建立健全政府重大决策的公众参与、专家论证、社会公示、社会听证、集体讨论决定等制度；改进和完善政绩考核办法，探索建立政府绩效评估体系和经济社会发展综合评价体系。

--进一步改进工作作风。牢固树立执政为民理念，把实现好、维护好、发展好最广大人民群众的根本利益作为一切工作的落脚点和出发点，真心实意地为人民群众办实事、做好事、解难事。深入开展调查研究，大力精简会议和文件，力戒空谈，克服官僚主义、形式主义和弄虚作假。坚定不移地抓好廉政建设和反腐败斗争，继续开展行风政风评议，切实纠正损害群众利益的行业不正之风，着力解决教育乱收费、医疗高收费等突出问题。贯彻实施《公务员法》，加强教育、管理、监督，努力建设一支政治坚定、业务精通、作风优良、人民满意的公务员队伍。

--进一步提高办事效率。强化行政监察，促进效能建设，缩短行政审批流程，加快办事节奏，降低社会各界到政府部门办事的制度性成本；加快电子政务建设，统一构建政府政务信息网络平台，实现市与县（市、区）及市级机关之间公文无纸化传输；推进行政审批网上协同办公，为企业、投资者和市民提供便捷的多功能服务。狠抓各项工作的落实，提倡现场办公和督促检查，加大督办力度，严格责任考核，着力解决办事拖拉、效率不高的问题，确保政令畅通。

各位代表，上饶正在加快发展的航程中奋进，我们对上饶的美好前景充满信心。站在新的历史起点上，我们要更加紧密团结在以胡锦涛同志为总书记的党中央周围，在省委、省政府和市委的领导下，一切为了上饶经济发展、一切为了社会全面进步、一切为了人民生活改善，抓紧每一天、走好每一步、做好每一件事，用我们的智慧和汗水书写新的历史篇章，为建设我们美好的上饶而阔步前进！
